# Comparison of SARS-CoV-2 Antibodies and Six Immunoassays in Pediatric and Adult Patients 12 Weeks After COVID-19

**DOI:** 10.7759/cureus.22195

**Published:** 2022-02-14

**Authors:** Imran Saglik, Alparslan Turkkan, Cansu Turan, Ates Kara, Halis Akalin, Beyza Ener, Ahmet Sahin, Edanur Yesil, Solmaz Celebi, Esra Kazak, Yasemin Heper, Emel Yilmaz, Muhammet Furkan Korkmaz, Esra Ture, Mustafa Hacimustafaoglu

**Affiliations:** 1 Department of Medical Microbiology, Bursa Uludag University Medical Faculty, Bursa, TUR; 2 Department of Public Health Sciences, Bursa Uludag University Medical Faculty, Bursa, TUR; 3 Department of Pediatric Infectious Diseases, Bursa Uludag University Medical Faculty, Bursa, TUR; 4 Department of Pediatric Infectious Diseases, Hacettepe University Medical Faculty, Ankara, TUR; 5 Department of Infectious Diseases and Clinical Microbiology, Bursa Uludag University Medical Faculty, Bursa, TUR; 6 Biochemistry and Clinical Biochemistry, Laboratory of Guven Tip, Bursa, TUR; 7 Department of Pediatric Infectious Diseases, Mersin City Hospital, Mersin, TUR; 8 Department of Pediatric Diseases, City Training and Research Hospital University of Health Sciences, Bursa, TUR; 9 Department of Pediatric Infectious Diseases, Bursa City Hospital, Bursa, TUR

**Keywords:** antibody persistence, adult, pediatric, sars-cov-2, serological assay, covid-19

## Abstract

Introduction

Severe acute respiratory syndrome coronavirus 2 (SARS-CoV-2)-specific humoral immune persistence has been proposed to be affected by patients’ characteristics. Moreover, available conflicting assay results are needed to be settled through comparative research with defined clinical specimens.

Methods

This prospective study investigated SARS-CoV-2-specific antibodies among 43 adults and 34 children at a mean of 12 weeks after the onset of COVID-19 symptoms using six serological assays and compared their performance. We used two Euroimmun (Euroimmun, Luebeck, Germany), two automated Roche Elecsys (Basel, Switzerland), and two rapid immuno-chromatographic Ecotest (Matrix Diagnostics, Assure Tech. (Hangzhou) Co., L, China) assays to investigate SARS-CoV-2 antibodies.

Results

The findings showed that the Roche Elecsys anti-S total test yielded the best positivity/sensitivity (children 94.1% and adults 93.0%; p = 0.877) while five immunoglobulin IgG targeting assays had similar positivity/sensitivity between children (88.2% to 94.1%) and adults (88.4% to 93.0%) (p > 0.05). Although IgM positivity was relatively low (p < 0.001), it was found in the majority of our pediatric and adult patients (67.6% and 86.0%, respectively; p = 0.098). SARS-CoV-2 S IgG titers were found to be higher among males in pediatric and adult groups compared to females (p = 0.027 and p = 0.041, respectively). Furthermore, we observed significantly higher antibody titers among pneumonia patients (p = 0.001).

Conclusion

Overall, we concluded SARS-CoV-2 antibody persistence over an average of 12 weeks after the onset of COVID-19 symptoms. While automated Roche Elecsys total antibody assays yielded the best sensitivity (> 90%) and five assays targeting IgG had acceptable performance. Patients with pneumonia and males have higher antibody titers. The effect of antibody persistence on re-infections should be monitored in longitudinal studies.

## Introduction

Accurate diagnosis of severe acute respiratory syndrome coronavirus 2 (SARS-CoV-2) infection is important for the management of the pandemic. Following the understanding of the structural proteins and antigenic properties of SARS-CoV-2, numerous serological diagnostic assays have been rapidly developed and implemented on a large scale. These assays vary by their technique (e.g., point-of-care-based lateral flow immunoassay-LFIA, enzyme-linked immunosorbent assay-ELISA, electrochemiluminescence immunoassay-ECLIA), revealed antibody class (e.g., immunoglobulin A (IgA), IgM, IgG or total), and targeted antigens (e.g., the nucleocapsid [N], subunit 1 [S1], or the receptor-binding domain [RBD] of the spike protein) [1,2]. Serological tests are highly specific but heterogeneous in their sensitivity for the diagnosis of coronavirus disease 2019 (COVID-19) [2-3]. Manufacturers usually report high sensitivity for their assays; however, heterogeneity is continued and the clinical sensitivity of a serological test may be inconstant, depending on its specifications, seroprevalence in the community, patient characteristics, and serum sampling time [3]. Previously reported studies are generally conducted in the acute infection period so data are insufficient to predict SARS-COV-2 immunoassays sensitivities in convalescence patients [4].

So far, the interest in the serology of SARS-CoV-2 has led researchers to compare different immunoassay results. Nevertheless, the comparison of antibody test results may be misleading because available serology tests may yield highly variable results due to their different characteristics [5-6]. In this respect, the World Health Organization (WHO) has issued an international standard (IS) to harmonize SARS-CoV-2 humoral immune response assessment and recommend reporting the results for binding antibody units (BAU). In this way, it is aimed to provide a standardization that would ensure the international validity of antibody test results [7].

SARS-CoV-2 antibody response may differ by infection severity or age and even not develop in some patients [2,8]. While seropositivity is observed among COVID-19 patients as early as five days after symptom onset, seroconversion rates may increase to > 90% by day 14. By the third week, seropositivity reaches the highest and then begins to decline, especially in mild cases [4,9-10]. Such a course is well-established by many performance studies of SARS-CoV-2 serologic assays on cohorts during the acute phase of the infection [2]. However, further studies are still needed to understand antibody responses and levels among patients following natural infection with SARS-CoV-2 [4]. Besides, SARS-CoV-2 serological tests were mostly evaluated in adults, so we have limited knowledge of pediatric patients. The studies that compared antibody levels of both children and adults are limited [11-12]. Recently, evidence of natural immunity to SARS-CoV-2 in children has drawn attention as a parameter that may help diagnose multisystem inflammatory syndrome in children (MIS-C or MIS-A in adults) [13]. Thus, understanding the dynamics and persistence of the different antibody responses after COVID-19 infection may also guide vaccine practices and the decision for booster doses [1,4,14].

The mutual dynamics of different commercial kits in a certain time period after infection may contribute to the interpretation of patients’ clinics. Ultimately, the aim of this study was to evaluate SARS-CoV-2 antibodies among adult and pediatric patients using six commercial immunoassays at the twelfth week after the COVID-19 infection and compare the performance of the assays.

## Materials and methods

The study complies with ethical issues, and ethical approval for this study was obtained from the Ethics Committee of Uludag University Faculty of Medicine (Date: 06/24/20, No: 2020-11/16).

Patients and serum samples

Seventy-seven SARS-CoV-2 reverse transcription-polymerase chain reaction (RT-PCR) confirmed COVID-19 patients (34 children and 43 adults) were included in this study between April and May 2020. We selected the subjects when the original Wuhan/D614G variant was circulating in Turkey and took the date of symptom onset as the reference time point. The patients were recruited to a SARS-CoV-2 PCR test within four days following their initial symptoms. Then, we obtained serum samples from the patients at the twelfth week after symptom onset.

Patients’ demographics, date of symptom onset, and symptoms were recorded. All patients had clinical findings consistent with COVID-19 and were categorized as pneumonic or non-pneumonic by their clinical and chest radiography (computed tomography (CT)/X-ray) findings following the WHO criteria [15]. Among 34 children, there were 19 without pneumonia (Group 1) and 15 with radiological evidence of pneumonia (Group 3). On the other hand, 24 of 43 adults were pneumonia (Group 3) and 19 were non-pneumonia (Group 4).

The RT-PCR tests were performed using Bio-Speedy SARS-CoV-2 double gene RT-PCR detection kits (Bioexen Ltd., Turkey) targeting the SARS-CoV-2-specific ORF1ab and N (nucleocapsid) gene fragment in a Rotorgene real-time PCR system (Qiagen, Germany) according to the manufacturer’s recommendations. Viral loads were measured through surrogate markers of cycle threshold (Ct) values for SARS-CoV-2-specific gene targets using RT-PCR assays applied to nasopharyngeal swab specimens.

Serological assays

Table 1 presents the general features of the six serological tests used in this study.

**Table 1:** Attributes the SARS-CoV-2 serologic assays ^1^Abbreviations: ELISA, enzyme-linked immunosorbent assay, ECLIA: electrochemiluminescence immunoassay, LFIA, lateral flow immunoassay, EUA: emergency use authorization, S1-RBD: S1 domain of the spike protein including the immunologically relevant receptor binding domain, N: Nucleocapsid. ^2^Usage of “pan” is for antibodies that will recognize different antigenic forms of a relating to all (or a large group of) protein species. ^3^Whole blood with taken by fingerstick blood collection card or sodium EDTA. ^4^S/Co: signal-to-cutoff, RU: Relatif unit, IU: International unit, BAU/mL; Baunding antibody unit/mL; EDTA: ethylenediaminetetraacetic acid. NA: not applicable.

Attribute	Euroimmun S IgG	Euroimmun QuantiVac S IgG	Roche Anti-N total	Roche Anti- S total	Ecotest pan^2^ IgM	Ecotest pan^2^ IgG
Assay principle	ELISA^1^	ELISA	ECLIA^1^	ECLIA	LFIA^1^	LFIA
Testing performed on	Manually	Manually	Automated (Cobas e 411)	Automated (Cobas e 411)	Manually	Manually
Antigen	S1-RBD^2^	S1-RBD	N-protein	S1-RBD	S and N^2^	S and N^1^
Antibody^3^	IgG	IgG	predominantly IgG, but also IgA, IgM	predominantly IgG, but also IgA, IgM	Pan IgM	Pan IgG
Sample type	Serum, plasma, dried blood spots^3^	Serum, plasma, dried blood spots^3^	Serum, plasma	Serum, plasma	Serum, plasma, whole blood^3 ^	Serum, plasma, whole blood^3 ^
Sample volume	10 µl	10 µl	20 μL	20 µL	5µl or one drop of blood	5µl or one drop of blood
EUA^1^ status	Approved	Approved	Approved	Approved	Approved	Approved
Interpretation	Qualitative	Quantitative	Qualitative	Quantitative	Qualitative	Qualitative
Result calculation^4^	Ratio (S/Co)	RU/mL, IU/mL, BAU/mL	Ratio (S/Co)	RU/mL,IU/mL, BAU/mL	Pos/neg	Pos/neg
Operational type	Batch	Batch	Continuous, random access	Continuous, random access		
Time to first result	3 hours	3 hours	18 min	18 min	15-30 min	15-30 min
Cut-off value (BAU/mL) Positive	≥1.1	≥35.2	≥1.0	≥0.8	Observation of test band	Observation of test band
Borderline	≥0.8 to <1.1	≥25.6 to <35.2	NA	NA	NA	NA

Enzyme-linked immunosorbent assay (ELISA) assays

We performed the Euroimmun Anti-SARS-CoV-2 ELISA IgG and anti-SARS-CoV-2 QuantiVac IgG assays (Euroimmun, Luebeck, Germany) on serum samples following the manufacturer’s instructions. The Euroimmun Anti-SARS-CoV-2 ELISA IgG results were evaluated qualitatively. Anti-SARS-CoV-2 QuantiVac provides a quantitative determination of specific IgG antibodies using a six-point calibration curve (measurement range 3.2‑384.0 BAU/ml). The test has been approved by an independent WHO reference material titled “First WHO International Standard Anti-SARS-CoV-2 immunoglobulin,” while BAU values are defined as equivalent international unit (IU) values [16].

Electrochemiluminescence immunoassay (ECLIA) assays

We performed the anti-N total (Roche, Elecsys SARS-CoV-2 anti-N total) and anti-S total (Roche, Elecsys, anti-SARS-CoV-2 S total) (Basel, Switzerland) assays on Cobas e 411 (Roche Diagnostics, Basel, Switzerland) through the ECLIA method per the manufacturer’s instructions. The measurement range of the Elecsys, anti-SARS-CoV-2 S total is 0.40-250.0 BAU/mL, and the test has also been approved by the said WHO reference material.

Food and Drug Administration (FDA) has approved Euroimmun and Roche SARS-CoV-2 antibody tests for use in patient diagnosis (EUA Authorized Serology Test Performance) [17].

Lateral flow immunoassay (LFIA) assays

The Ecotest (Matrix Diagnostics, Assure Tech. (Hangzhou) Co., L, China) is a lateral flow chromatographic immunoassay for the qualitative detection of antibodies against SARS-CoV-2 in humans. This test contains pan SARS-CoV-2 antigen for the detection of human IgM or IgG as the capture, and results are interpreted 15 min after testing by experienced healthcare staff. The test has also been authorized by FDA under EUA for use [18].

Statistical analysis

We conducted all statistical analyses using SPSS (IBM Corp., Armonk, NY). While continuous variables were shown as mean ± SD, we presented categorical variables as percentages. We analyzed antibody titers based on the results of two quantitative tests (Euroimmun QuantiVac IgG and Roche anti-S total). The results of the other tests were interpreted only qualitatively. Besides, we used Cochran’s Q test and McNemar’s test to compare continuous non-parametric variables. Moreover, we compared the categorical variables using Fisher’s exact test and continuity correction. We considered a p-value of <0.05 to be statistically significant in all statistical analyses.

We concluded borderline findings only in five serum samples with Euroimmun assay and accepted them as positive for the analysis [19-20].

## Results

The mean age was 11 years in children (95% CI 9.2-12.8; range = 0.4-17.5 years) and 39.2 years in adults (95% CI 35.7-42.7; range = 18.4-79.6 years). Antibody response was detected with at least one of the tests (73 (94.8%)) of the serum samples from 77 patients while 55 patients (71.4%) had antibody response at the end of all the tests. Four patients (two adults and two children) without any antibody response by any test had no clinical findings of pneumonia (Table 2).

**Table 2:** Comparison of negative results of assays Pos: positive, neg: negative

Total n=22	Euroimmun S IgG	Euroimmun QuantiVac S IgG (mean BAU/mL)	Roche Anti-N total	Roche Anti-S total (mean BAU/mL)	Ecotest pan IgM	Ecotest pan IgG
n=13	pos	pos (278.4)	pos	pos (203.8)	neg	pos
n=4	neg	neg (3.2)	neg	neg (0.4)	neg	neg
n=2	neg	neg (3.2)	pos	pos (12.9)	pos	neg
n=1	borderline	neg (23.3)	pos	pos (20.3)	pos	pos
n=1	borderline	neg (23.6)	pos	pos (26.9)	pos	neg
n=1	neg	neg (3.2)	neg	neg (0.4)	pos	neg

As presented in Table 3, pediatric and adult patient groups showed similar characteristics regarding sex distribution, disease severity, and developing pneumonia. Moreover, SARS-CoV-2 IgG or total antibody positivity in serum samples was similar in both children (lowest: 88.2% - highest: 94.1%) and adults (lowest: 88.4% - highest: 93.0%). The mean positivity rate of IgM antibodies was found to be higher in adults (86%) than in children (67.6%), but the difference was not significant (p = 0.098). Quantitative results of Euroimmune QuantiVac IgG and Roche S total assays yielded that the SARS-CoV-2 antibody titers of adult patients were higher than those of children, but the differences were not significant (p = 0.686 and p =0.877, respectively) (Table 3).

**Table 3:** Comparison of the patients’ characteristics and assays results Neg: negative, Pos: positive ^1^Rt-PCR Ct: Reverse transcription-polymerase chain reaction cycle threshold; Total n=46 (pediatric n=21, adult n=25) ^2^Fisher’s exact test ^3^Continuity correction

	Total n=77	Pediatric n=34 (44.2%)	Adult n=43 (55.8%)	P
Patients characteristics n, (%)				
Sex, male	44 (57.1)	23 (67.6)	21 (48.8)	0.154
Pneumonia	39 (50.7)	15 (44.1)	24 (55.8)	0.192^3^
Respiratory distress	5 (6.5)	4 (11.8)	1 (2.3)	0.164^2^
Baseline Rt-PCR Ct^1 ^valueMean ± SD	25.9±6.9	28.3±6.6	24.6±6.8	0.078
Antibody titers Mean ± SD (BAU/mL)				
Euroimmun QuantiVac S IgG	230.1 ±137.4	222.9 ±139.8	235.8 ±136.8	0.686
Roche Anti-S total	170.2 ±99.1	168.2 ±102.3	171.8 ±97.7	0.877
Antibody positivity n, (%)				
Euroimmun S IgG				
Neg	7 (9.1)	4 (11.8)	3 (7.0)	0.693^2^
Pos (sensitivity)	70 (90.9)	30 (88.2)	40 (93.0)	
Euroimmun QuantiVac S IgG				
Neg	9 (11.7)	4 (11.8)	5 (11.6)	1.000^2^
Pos (sensitivity)	68 (88.3)	30 (88.2)	38 (88.4)	
Roche anti-N total				
Neg	6 (7.8)	2 (5.9)	4 (9.3)	0.689^3^
Pos (sensitivity)	71 (92.2)	32 (94.1)	39 (90.7)	
Roche anti-S total				
Neg	5 (6.5)	2 (5.9)	3 (7.0)	1.000^2^
Pos (sensitivity)	72 (93.5)	32 (94.1)	40 (93.0)	
Ecotest pan IgG				
Neg	9 (11.7)	4 (11.8)	5 (11.6)	1.000^2^
Pos (sensitivity)	68 (88.3)	30 (88.2)	38 (88.4)	
Ecotest pan IgM				
Neg	17 (22.1)	11 (32.4)	6 (14.0)	0.098^3^
Pos (sensitivity)	60 (77.9)	23 (67.6)	37 (86.0)	

The most sensitive SARS-CoV-2 antibody assay was the Roche anti-S total with 93.5% sensitivity, followed by the Roche anti-N total with 92.2% sensitivity. Although not statistically significant, both Roche assays measuring total antibodies (IgG, IgM, and IgA) through the ECLIA method showed higher sensitivity than the other assays (Table 3). However, five assays for only IgG or total antibodies (Roche anti-N total, Roche anti-S total, Euroimmune anti-IgG, Euroimmun QuantiVac IgG, and Ecotest IgG) had statistically similar sensitivity, according to the results of both Cochran’s Q test (p = 0.074-0.264 for all) and McNemar’s test (p = 0.125-1.000 for all). All patients with Ecotest had 88.3% IgG and 77.9% IgM positivity. The positivity of the Ecotest pan IgM test was significantly lower than all other tests (p < 0.001).

Regarding sex, antibody positivity rates were found to be similar between male and female patients by all assays. However, in quantitative measurements, the mean antibody titers measured by Roche anti-SARS-CoV-2 S (192.7 ± 96.6 BAU/mL) and Euroimmun anti-SARS-CoV-2 QuantiVac IgG (267.9 ± 133.2 BAU/mL) assays were significantly higher among males than females (p = 0.004 and p = 0.050, respectively) (Table 4).

**Table 4:** Comparison of antibody positivity rates and titers between females and males in pediatric and adult patients

		Total		Pediatric		Adult	
		n=77	p	n=34	p	n=43	p
SARS-CoV-2 RT-PCR Ct value Mean ± SD	female	24.6±6.5	0.227	22.9±0.2	0.003	25.0±7.2	0.636
	male	27.1±7.1		30.1±6.7		23.8±6.3	
The time of collecting serum samples after initial symptoms Mean ± SD (week)	female	12.1±1.4	0.569	11.9±1.8	0.268	12.2±2.1	0.466
	male	12.6±1.3		12.5±1.9		12.8±1.5	
Antibody titers Mean ± SD (BAU/mL)							
Euroimmun QuantiVac IgG	female	179.0±128.1	0.004	150.3±115.8	0.027	194.5±133.9	0.041
	male	267.8±133.2		257.6±139.1		279.0±128.9	
Roche Anti-S total	female	148.3±97.3	0.050	150.0±105.6	0.325	147.4±95.4	0.095
	male	192.6±96.6		188.5±99.5		197.2±95.7	
	male	12.6±1.3		12.5±1.9		12.8±1.5	
Antibody positivity, positive n (%)							
Euroimmun Anti-S IgG	female	29 (87.9)	0.689	10 (90.9)	1.000	19 (86.4)	0.248
	male	41 (93.2)		20 (87.0)		21 (100.0)	
Euroimmun QuantiVac Anti-S IgG	female	28 (84.8)	0.486	10 (90.9)	1.000	18 (81.8)	0.370
	male	40 (90.9)		20 (87.0)		20 (95.2)	
Roche Anti-N total	female	29 (87.9)	0.425	11 (100.0)	0.819	18 (81.8)	0.127
	male	42 (95.5)		21 (91.3)		21 (100.0)	
Roche Anti-S total	female	30 (90.9)	0.739	11 (100.0)	0.819	19 (86.4)	0.248
	male	42 (95.5)		21 (91.3)		21 (100.0)	
Ecotest IgG total	female	28 (84.8)	0.645	18 (81.8)	0.370	10 (90.9)	1.000
	male	40 (90.9)		20 (95.2)		20 (87.0)	
Ecotest IgM total	female	26 (78.8)	1.000	7 (63.6)	1.000	19 (86.4)	1.000
	male	34 (77.3)		16 (69.6)		18 (85.7)	

IgG antibodies were positive in all pneumonic patients (n = 39). Comparing the assays targeting IgG and total antibodies, we found that patients with pneumonia had significantly higher positivity rates (Euroimmun IgG, Euroimmun QuantiVac IgG, Roche anti-N total, and Ecotest IgG) when compared to those without pneumonia (Table 5). In addition, anti-N test and anti-S assays showed 100% agreement. Moreover, quantitative antibody titers (with Euroimmune Anti-SARS-CoV-2 QuantiVac IgG and Roche Anti-SARS-CoV-2 S total tests) were found to be higher in patients with pneumonia (p = 0.001) (Table 5).

**Table 5:** Comparison of positivity and titers of antibody in patients with and without pneumonia Pos: positive, neg: negative

Antibody positivity n (%)	n (%)	Pneumonia		p
		No, n (%)	Yes, n (%)	
Euroimmun S IgG	neg 7 (9.1)	7 (17.5)	0 (0.0)	0.023
	pos 70 (90.9)	33 (82.5)	37 (100.0)	
Euroimmun QuantiVac S IgG	neg 9 (11.7)	9 (22.5)	0 (0.0)	0.007
	pos 68 (88.3)	31 (77.5)	37 (100.0)	
Roche anti-N total	neg 6 (7.8)	6 (15.0)	0 (0.0)	0.043
	pos 71 (92.2)	34 (85.0)	37 (100.0)	
Roche anti-S total	neg 5 (6.5)	5 (12.5)	0 (0.0)	0.078
	pos 72 (93.5)	35 (87.5)	37 (100.0)	
Ecotest pan IgG	neg 9 (11.7)	9 (22.5)	0 (0.0)	0.007
	pos 68 (88.3)	31 (77.5)	37 (100.0)	
Ecotest pan IgM	neg 17 (22.1)	12 (30.0)	5 (13.5)	0.142
	pos 60 (77.9)	28 (70.0)	32 (86.5)	
Antibody titers Mean ± SD (BAU/mL) n=77		n=38	n=39	
Euroimmun QuantiVac S IgG		182.2±142.9	281.9±111.3	0.001
Roche S total		136.6±107.7	213.7±69.7	0.001

Considering baseline viral RT-PCR Ct findings, we could reach only 46 patients' Ct data in the laboratory records. Accordingly, the mean viral Ct value of these patients was 25.8 ± 6.9. Besides, mean Ct values were similar in female (24.6 ± 6.5) and male (27.1 ± 7.1) patients (p = 0.227) and in children (Ct = 28.2 ± 1.4) and adult (Ct = 25.9) patients (p = 0.12). When it comes to quantitative assays, baseline Ct values were found to be lower (supporting higher viral load) in antibody-positive patients (24.2 ± 5.7 (n = 39) and 35.1 ± 5.6 (n = 7), p < 0.001 for Euroimmun QuantiVac IgG positive (n) and negative (n) patients, respectively; 24.4 ± 5.5 (n = 41) and 36.1 ± 2.3 (n = 5), p < 0.001 for Roche anti-S total positive (n) and negative (n) patients, respectively).

## Discussion

The focus of the present study was on assessing antibody persistence characteristics/reasons and serological assays performance among adult and pediatric patients at the twelfth week after COVID-19 infection. The results revealed highly antibody positivity of serum samples to different antigens of SARS-CoV-2 (anti-S total = 93.5%, anti-N total = 92.2%, anti-S IgG = 88.3-90.9%, pan IgG = 88.3%, and pan IgM = 77.9%) in both children and adults. While the positivity rates and titers of antibodies did not differ significantly between pediatric and adult patients, it was noteworthy that the antibody titers were higher in male patients and patients who developed pneumonia during active COVID-19 infection. It appears that baseline Ct values were found to be lower (supporting higher viral load) in antibody-positive patients. Besides, five of the six different immunoassays in our study (except for the Eco pan IgM test targeting IgM) had similar sensitivity and ability to detect antibodies.

It is known that IgG antibodies to SARS-CoV-2 S1-RBD are strongly correlated with neutralizing antibody titers, therefore, antibody levels may help predict the protectivity during re-infection (264 BAU/mL anti-spike IgG may provide 80% protection from symptomatic infection) [6,21]. In this study, we measured antibody titers against Spike S1-RBD using two quantitative assays - Euroimmun QuantiVac IgG and Roche anti-S total - calibrated with WHO IS serum. The results of these two tests were found to be statistically similar (p = 0.125). However, when comparing the titers of these two assays, two factors should be remembered. First, one of the assays measures only IgG while the other measures total IgG/M/A titers. IgM antibodies may be durable at the twelfth week and provide an additional advantage to total antibody assays (even if the IgG response is absent or lower) [22]. Second, as in our study, differences in the upper quantitation limits of the assays (384 and 250 BAU/ml) may affect the results. In the present study, antibody titers were found to be >250 BAU/ml in 37 cases (48.1%) (>384 BAU/ml in 18 cases (23.7%)) with the Euroimmune QuantiVac S IgG test while being >250 BAU/ml in 38 cases (49.3%) with the Roche anti-S total test. Ultimately, the characteristics of serological assays (method, used antigens and target immunoglobins isotypes, as well as quantitation/detection limit) should be considered when comparing, evaluating, and reporting antibody levels. In our opinion, the harmonization and standardization between two assays are partially satisfied but inadequate to compare antibody levels in patients or establish a precise for immunity.

The previous researchers compared SARS CoV-2 antibody assays’ performance mostly in the acute infection period [1,5]. Accordingly, Haselmann V et al. reported that the Roche and Euroimmun immunoassays had 92.3% and 96.2-100% diagnostic sensitivity and 100% specificity in acute COVID-19 infection, respectively [20]. Kittel et al., comparing six commercial antibody tests (including the Euroimmune IgG test), found the Roche total assay to be the most sensitive in their study [5]. When it comes to our study, we presented data from six immunoassays from three vendors and methods. The Roche anti-N and anti-S total tests, which use the fully automated high-throughput ECLIA method, showed better performance, although we could not detect a significant difference between them. Overall, Roche antibody tests have the capability to detect the total antibody response with an automated system, which may minimize redundant usage and laboratory errors and explain the increase in sensitivity. Hence, we can confidently assert that serological assays in our study targeting IgG (sensitivity ≥88.0%) exhibit good performance, detect seropositivity at a high rate, and have no difference in overall sensitivity 12 weeks after COVID-19 infection (p>0.05). It is an advantage that Euroimmun Quantivac IgG provides quantitative results with similar performance to a fully automated system. However, with the micro ELISA method, the loss of the kit due to the use of controls (two wells) and calibrations (six wells) in the Euroimmun Quantivac IgG may restrict the use of the assay. When evaluating commercial LFIA (Ecotest), we observed more intense streaking in cases with high quantitative antibodies and found no borderline results, as well as being easily interpreted. Thus, we think that the performance of Ecotest with enables rapid results is acceptable 12 weeks after natural infection and may be useful in centers with limited facilities.

Some studies have investigated the sensitivity of SARS-CoV-2 serological assays are based on antigens used in the assay. For example, Fenwick et al. reported that antibody responses to S1 and N proteins were equally sensitive in antibody detection in the acute-infection-phase samples. However, in the post-infection phase, antibody response to N protein appears to wane over (e.g., months), although anti-S antibody responses persisted. Besides, the authors observed lower sensitivity due to the decay of N antibodies on convalescent sera taken two months after the symptoms [23]. Jacot et al. reported no differences between N-based and S-based assays during the first 38 days of the symptoms [24]. In this study, although the difference was not significant, we detected the Roche Anti-S total test (93.5%) had slightly better sensitivity than the Roche anti-N total test (92.2%) 12 weeks after COVID-19 infection. Especially, perfect agreement in pneumonia patients between Roche anti-N and anti-S tests suggests that such an agreement between the assays targeting both antigens is pretty acceptable even during the convalescence of patients with pneumonia.

Antibody responses to other human coronaviruses were reported to wane over time. For instance, antibody responses to endemic human α- and β-coronaviruses can last only 12 weeks [25]. In COVID-19 cases, seropositivity reaches nearly 100% in the third week and may decrease then [4,22]. Nevertheless, antibody titers may remain negative in about 5% of symptomatic PCR-positive patients [26]. SARS-CoV-2 antibody response may decrease or patients may become seronegative over time. Lyer et al. reported that IgG antibodies to SARS-CoV-2 RBD were little to no decrease over 75 days since symptom onset despite the rapid decline of IgM responses (the median time to seroreversion for IgM was 48.9 days) [6]. In another study, IgM antibodies were detected at 12.8%, while IgG antibody positivity was 82.9% in convalescent patients with confirmed SARS-CoV-2 infection a year ago [22]. In our study, IgM positivity was 77.9% (via Ecotest IgM) 12 weeks after the onset of symptoms, and it was significantly lower than IgG positivity such as expected (p < 0.001). A study with a broad cohort demonstrated that the follow-up seropositive patients, whose 12.4% were negative when retested within 0 to 30 days, became seronegative at 18.4% retested after more than 90 days [27]. In this study, antibody negativity was 6.5-11.7% (children: 5.9-11.8% and adults: 7.0-11.6%) at the twelfth week after the symptom onset. We do not know whether their test results were negative from the beginning or whether they became negative within 12 weeks. Our study was not conducted longitudinally, so we could not interpret the course of antibody titers.

Children generally developed milder forms of the viral disease, which may be due to their relatively immature immune systems not causing exacerbated inflammation response [28]. Most children with SARS-CoV-2 infection are either asymptomatic or exhibit mild symptoms in contrast to adult patients [29]. A study involving approximately 2000 children and adolescents reported that 46.2% of the seropositive children were asymptomatic and that their antibody titers were low compared to those of the adults [29-30]. The relevant literature, hosting only a few studies on this subject, shows evidence that SARS-COV-2 antibodies (anti-S RBD IgG, anti-N IgG, and neutralizing antibodies) are fewer in children when compared to adults [11-12]. In this study, we comparatively explored child and adult antibody levels. Although the antibody titers measured in children were lower than in adults, the differences were found to be statistically similar. Several conditions may have caused this result. The relevant studies clearly showed that patients with severe clinical course COVID-19 often have higher antibody levels consistent with our work [9,31]. First, the similarity (p=0.192) of disease severity (e.g. pneumonia rates) between our pediatric (44.1%) and adult (55.8%) groups may have led us to find antibody titers at similar levels. Second, Dailey et al. reported that antibody response is often weaker among immunocompromised patients [12]. Therefore, similar antibody responses between children and adults in our study may have been related to all patients with similarly normal immunity.

Antibody levels, namely, the humoral immune response, may vary by the severity of COVID-19 infection as mentioned above [9,31]. In a study, hospitalized patients with severe infection produced a strong antibody response to SARS-CoV-2 with a high correlation between different viral antigens (S and N), and only a few asymptomatic subjects developed antibodies at detectable levels [32]. In another longitudinal study, SARS-CoV-2 IgG antibodies (anti-N and anti-S by the ECLIA method) were found to be significantly lower in asymptomatic cases compared to symptomatic cases in the first-year convalescent serum samples of 473 cases [33]. In line with the previous research, we found antibody positivity in both children and adults to be significantly higher in cases with pneumonia than in mild cases. Moreover, we found that the clinical course of pneumonia (more lung involvement) was positively associated with higher levels of anti-S1-RBD titers. Sun et al. reported a positive correlation between IgG antibodies and disease severity, but it was not the case for IgM antibodies [34]. Similarly, in our study, IgM positivity did not significantly differ between patients with pneumonia (86.5%) and those without pneumonia (70.0%) (p=0.142).

The clinical course of COVID-19 may differ by sex, and immunological antibody responses between males and females have also been the subject of research. In their study, exploring antibody response between 21-212 days after the symptom onset, Markmann et al. reported that higher neutralizing antibody titers were significantly associated with male sex; found robust antibody durability up to six months, as well as a significant positive association between the magnitude of the neutralizing antibody response and male sex [35]. Similarly, in our study, antibody levels with the Euroimmune QuantiVac IgG test were found to be higher in both pediatric and adult males. These results may help explain the factors affecting the overall disease course between males and females and allow us to make some speculations about the severe course of the disease among males. The results of the other quantitative test, Roche anti-S total, yielded higher antibody levels in both pediatric and adult males, but the difference was not statistically significant, which may be because the upper quantitation limit of the relevant test (250 BAU/ml) is lower than that of the Euroimmune QuantiVac IgG test (384 BAU/mL).

SARS-CoV-2 IgG and IgM antibodies have been widely used to assist various clinical diagnoses. The MIS-C represents a post-infectious complication and/or antibody-related hyperinflammatory complication (three- to four-week lag) rather than acute infection in some children. Many affected children have negative PCR testing for SARS-CoV-2 but have significantly higher antibody titers [13]. In this respect, detecting seropositivity accurately may contribute to the diagnosis of MIS-C in 12 weeks. If about 70% RT-PCR positivity (sensitivity) is taken as reference in acute infection in some cases; IgM, IgG, or total SARS CoV-2 antibody response between 77.9% and 93.0% in COVID-19-positive cases in our study can be accepted as evidence of high rates of seropositivity from the tests [13,36].

There are also some studies that nasopharyngeal viral load affected the infection severity during SARS-CoV-2 infection [37]. Besides, there may be an association between viral load or antibody response or titers, although little research previously attempted to uncover it. While some studies reported that higher S-antibody levels are associated with a faster decreased viral load and earlier antibody response [38-39], patients without seroconversion show the lowest viral loads at the other end of the spectrum [39]. It seems that the kinetics of the humoral immune response predicts the speed of viral elimination; for example, the earlier antibody response was associated with a faster viral clearance. Confusingly reported that patients who did not seroconvert were found to have higher cycle threshold values of RT-PCR (38.0 vs. 28.0) and a shorter time to viral clearance. Jin et al. reported that prolonged viral shedding is associated with higher levels of S IgG, probably reflecting a higher release of antibodies due to prolonged exposure to the virus [40]. In our study, following the previous findings, patients with negative S-antibody response as a result of two tests (Euroimmune QuantiVac IgG and Roche anti-S total) had significantly higher RT-PCR mean Ct values ​​(35.1, 36.1, respectively) than those with positive antibody response ​​(24.2, 24.4, respectively) (p < 0.001). However, it should be remembered that received antiviral agents, immunomodulators, and sampling time for RT-PCR may have affected these parameters [39].

The present study is deemed to have several strengths. Most studies focused on only acute-stage patients since the very first emergence of the pandemic, and the findings pertinent to antibody levels of pediatric groups are still lacking [29]. We performed the study with subjects with similar clinical severity distribution, which gave us a chance to investigate antibody responses of different age groups with different clinical courses. Moreover, we took the date of the onset of symptoms as the reference time point, which might be a more accurate reference point than RT-PCR [5].

On the other hand, the present study is not free of a few limitations. In this study, we assessed antibody persistence and assay performance and recorded the detailed disease courses of the patients but did not discuss these issues in detail. Moreover, we included only convalescent patients after COVID-19 but excluded negative patients and different variants of SARS-CoV-2. Finally, we did not design the study as a longitudinal one and could not comment on the durability of the antibody response over time.

To sum, the present study provides valuable highlights on the serological assays used to analyze immunity against SARS-CoV-2 and antibody persistence among convalescent COVID-19 individuals. Furthermore, we showed that IgG class-based assays for SARS-CoV-2-specific antibodies have >85% positivity/sensitivity after 12 weeks in adult and pediatric patients, and the IgM antibodies may be detected lower due to the serum sampling time. Severe (e.g., pneumonia) COVID-19 is an important factor for persisted, higher antibodies in both children and adults. Thus, a considerable gap in knowledge regarding long-term antibody kinetics after natural infections - particularly various variants of SARS-CoV-2-waits to be clarified by longitudinal serological studies. We hope to gain a better-determined duration of immunity and its effect on reinfections in the future.

## Conclusions

To sum, the present study provides valuable highlights on the serological assays used to analyze immunity against SARS-CoV-2 and antibody persistence among convalescent COVID-19 individuals. Furthermore, we showed that IgG class-based assays for SARS-CoV-2-specific antibodies have >85% positivity/sensitivity after 12 weeks in adult and pediatric patients, and the IgM antibodies may be detected lower due to the serum sampling time. Severe (e.g., pneumonia) COVID-19 is an important factor for persistent, higher antibodies in both children and adults. Thus, a considerable gap in knowledge regarding long-term antibody kinetics after natural infections, particularly various variants of SARS-CoV-2, waits to be clarified by longitudinal serological studies. We hope to gain a better-determined duration of immunity and its effect on reinfections in the future.
